# Efficacy and Safety of Polymyxins for the Treatment of *Acinectobacter baumannii* Infection: A Systematic Review and Meta-Analysis

**DOI:** 10.1371/journal.pone.0098091

**Published:** 2014-06-09

**Authors:** Qianqian Liu, Wenzhang Li, Yulin Feng, Chuanmin Tao

**Affiliations:** 1 Department of Laboratory Medicine, West China Hospital, Sichuan University, Chengdu, Sichuan Province, China; 2 Department of Cardiology, First Affiliated Hospital of Chengdu Medical College, Chengdu, Sichuan Province, China; 3 Department of Respiratory Medicine, West China Hospital, Sichuan University, Chengdu, Sichuan Province, China; University of Malaya, Malaysia

## Abstract

**Background:**

Multi-drug resistance among *Acinetobacter baumannii* increases the need for polymyxins. We conducted a meta-analysis aimed to assess the efficacy and safety of polymyxins for the treatment of *Acinetobacter baumannii* infection.

**Methods:**

We searched PUBMED, EMBASE, the Cochrane Central Register of Controlled Trials (CENTRAL), CNKI, Chinese Biomedical Literature Database up to November 1, 2013, to identify published studies, and we searched clinical trial registries to identify completed unpublished studies. Randomized controlled trials and cohort studies were considered for inclusion. Data were extracted on clinical response, microbiological response, mortality, length of stay and adverse events.

**Results:**

12 controlled studies, comparing 677 patients, were included. Although clinical (odds ratio 1.421, 95% confidence interval 0.722–2.797) and microbiological (OR 1.416, 95% CI 0.369–5.425) response rates favored the polymyxins group, these differences were not significant. Treatment with polymyxins vs. controls did not affect hospital mortality (OR 0.506, 95% CI 0.101–2.536), lengths of hospital stay (standard mean difference −0.221, 95% CI 0.899–0.458) or nephrotoxicity (OR 1.192, 95% CI 0.436–3.261). The combination of polymyxins with other antibiotics achieved similar clinical response rates to its monotherapy regimen (OR 0.601, 95% CI 0.320–1.130).

**Conclusions:**

Our results suggest that polymyxins may be as safe and as efficacious as standard antibiotics for the treatment of *A. baumannii* infection. There is no strong evidence that combination regimen of polymyxins is superior to monotherapy regimen.

## Introduction

Being a non-fermentative Gram-negative coccobacillus, *Acinetobacter baumannii* has become an increasingly notorious pathogen of healthcare associated infection in recent years. According to the data from a USA 7-year national surveillance project that detected 24179 cases with healthcare associated bacteremia, *A. baumannii* was found to be the 10th most frequent pathogen and crude mortality ranges from 30% to 50% in patients with *A. baumannii* bacteremia [1]. This pathogen can lead to different types of infections such as respiratory tract infection, bloodstream infection, skin and soft tissue infections, urinary tract infections, and meningitis. *A. baumannii* associated infection assumed the tendency of increase year by year [2]. The enhanced environmental resilience and multi-drug resistance renders *A. baumannii* a successful colonizing pathogen [3] and usually results in high treatment failure [4]. The Infectious Diseases Society of America has been trying hard to address these challenges [5], but to little avail. It has a long way to go for the development of truly powerful antibiotics. Although limited help is in sight from the current drug discovery pipeline, the re-use of polymyxins enable us to see the dawn [6].

Polymyxins, discovered in 1940s, are a group of polycationic peptide antibiotics, exhibiting potent efficacy against most gram-negative bacteria [7] [8]. Among all the five chemical compounds (A–E) of polymyxins, only polymyxin B and E (colistin) are used clinically, with structure difference of one amino acid. Since 1970s they were practically abandoned in most patient populations owing to reports of severe adverse events. Yet, in the recent decade, multi-drug resistant (MDR) Gram-negative infections, especially in critically ill patients, prompt the reviving of polymyxins, which has proven less toxicity than previous reports [9]–[11]. Although the available evidence suggests that polymyxins based therapies can be effective for the treatment of *A. baumannii* infection, whether polymyxins-based therapies are more or less effective than alternative therapies is still in debate.

Meanwhile, along with the re-emergence of polymyxins in the treatment of *A. baumannii* infection, its best regimen remains unsettled. It is especially hard to judge when comparing polymyxins monotherapy with combination therapy. Several in vitro and animal studies have demonstrated polymyxins’ synergistic activity with other agents particularly rifampicin [12]–[15]. By altering membrane permeability, polymyxins may facilitate rifampicin’s entry into the bacterial cell and then bring about enhanced antibacterial activity [16] [17]. Can enhanced bactericidal activity translate into improved clinical outcomes? It is a question to be defined. On the other hand, the superiority of combination regimen has been questioned with regard to the probability of increased toxicity and cost. Therefore, more data are needed to clarify the role of different regimens in the treatment of *A. baumannii* infection. The aim of our study is to systematically evaluate the efficacy and safety of polymyxins in the treatment of *A. baumannii* infection and to compare monotherapy and combination treatment regimens. This report follows the PRISMA (Preferred Reporting Items For Systematic Reviews and Meta-Analyses) statement [18].

## Methods

### Search Strategy

The following databases from their inception until November 1, 2013 were searched: PUBMED, EMBASE, the Cochrane Central Register of Controlled Trials (CENTRAL), CNKI, and Chinese Biomedical Literature Database. To identify relevant completed studies that were unpublished, we searched relevant web sites (http://www.clinicalstudyresults.org and http://www.clinicaltrials.gov). The main search concepts were “*baumannii* and colistin”, “*baumannii* and polymyxin”, “*baumannii* and colistimethate”. Furthermore, the reference lists of reports identified by this search strategy were also searched to select relevant articles.

### Selection Criteria

Studies were considered eligible for inclusion if they were randomized controlled trials (RCT) or cohort studies with designs comparing the efficacy and safety of polymyxins against other antimicrobial agents for the treatment of *A. baumannii* infection. We also try to summarize the information on the efficacy and safety of polymyxins monotherapy in comparison with the combination treatment for *A. baumannii* infection.

Experimental trials in animals, trials focusing on pharmacokinetic or pharmacodynamic variables, trials referring only to the in-vitro activity of polymyxins and incomplete unpublished studies were excluded from the meta-analysis. No language restrictions were applied.

### Data Abstraction and Quality Assessment

The abstraction of data was conducted by two independent investigators. Discrepancies were resolved by discussion and simultaneous reference to the relevant literatures. The following variables were collected from studies: title, primary author’s name, year and source of publication, country of origin, study design, baseline characteristics of the study population (sample size, age, sex, disease, severity of illness), type and dose of polymyxins administered, coadministration of other antibiotics, duration of the treatment, outcomes (clinical and microbiological response, length of hospital stay, in-hospital mortality and reported toxicity). If data concerning the outcome were not displayed in the article, the investigators would contact the primary author in an attempt to obtain the missing data.

The main efficacy outcome of interest was clinical response. Other efficacy outcomes were microbiological response, in-hospital mortality and in-hospital stay length. The main safety outcome of interest was nephrotoxicity. Other safety outcomes were neurotoxicity. Clinical response was defined as complete or partial remission of the signs and symptoms of infection by the end of therapy [19]. Microbiological response was defined as negative of culture result at the end of therapy [19]. In patients with normal renal function (serum creatinine level <1.2 mg/dl), nephrotoxicity was defined as a serum creatinine value of >2 mg/dl, as a reduction in the calculated creatinine clearance of 50% relative to the value at the initiation of antibiotic therapy, as initiation or as a decline in renal function that prompted renal replacement therapy [20]. In patients with pre-existing renal dysfunction, nephrotoxicity was defined as an increase of 50% from the baseline creatinine level, as a reduction in the calculated creatinine clearance of 50% relative to the value before polymyxins therapy was initiated [20]. Neurotoxicity was defined as any of the following: seizures, encephalopathy, neuromuscular blockade and apnea [20].

We evaluated the quality of RCTs according to Jadad scale [21], This widely used scale assessed the reporting of studies on the basis of three fundamental methodological criteria: the method of randomization, adequacy of double-blinding and the completeness of follow-up. The minimal and maximal scores for an included study were 1 and 5, respectively. Studies with score less than three were considered as low quality and were excluded from this meta-analysis.

The Newcastle-Ottawa scale (NOS) score was determined to assess the quality of cohort studies included in the meta-analysis [22]. Studies with a NOS score <3 were classified as poor quality and were excluded from this meta-analysis.

### Statistical Analysis

For each study, the between-study heterogeneity was assessed by the χ^2^ based Q statistics and I^2^ test. Heterogeneity was considered as I^2^>50%. For studies with no event of interest in a treatment group, 1.0 was then added to all cells. Binary outcomes results were expressed as odds ratios (ORs), and continuous outcomes results were expressed as standard mean difference (SMD) between 2 groups. All of the data from each study used either fixed effects (Mantel–Haenszel’s method) or random-effects (DerSimonian and Laird’s method) models according to the heterogeneity result. Begg’s and Egger’s tests were used to test the possible publication bias. All of the above analyses were performed by STATA 12.0 software, using two-sided P values.

## Results

### Flow of Included Studies

**Figure 1:**
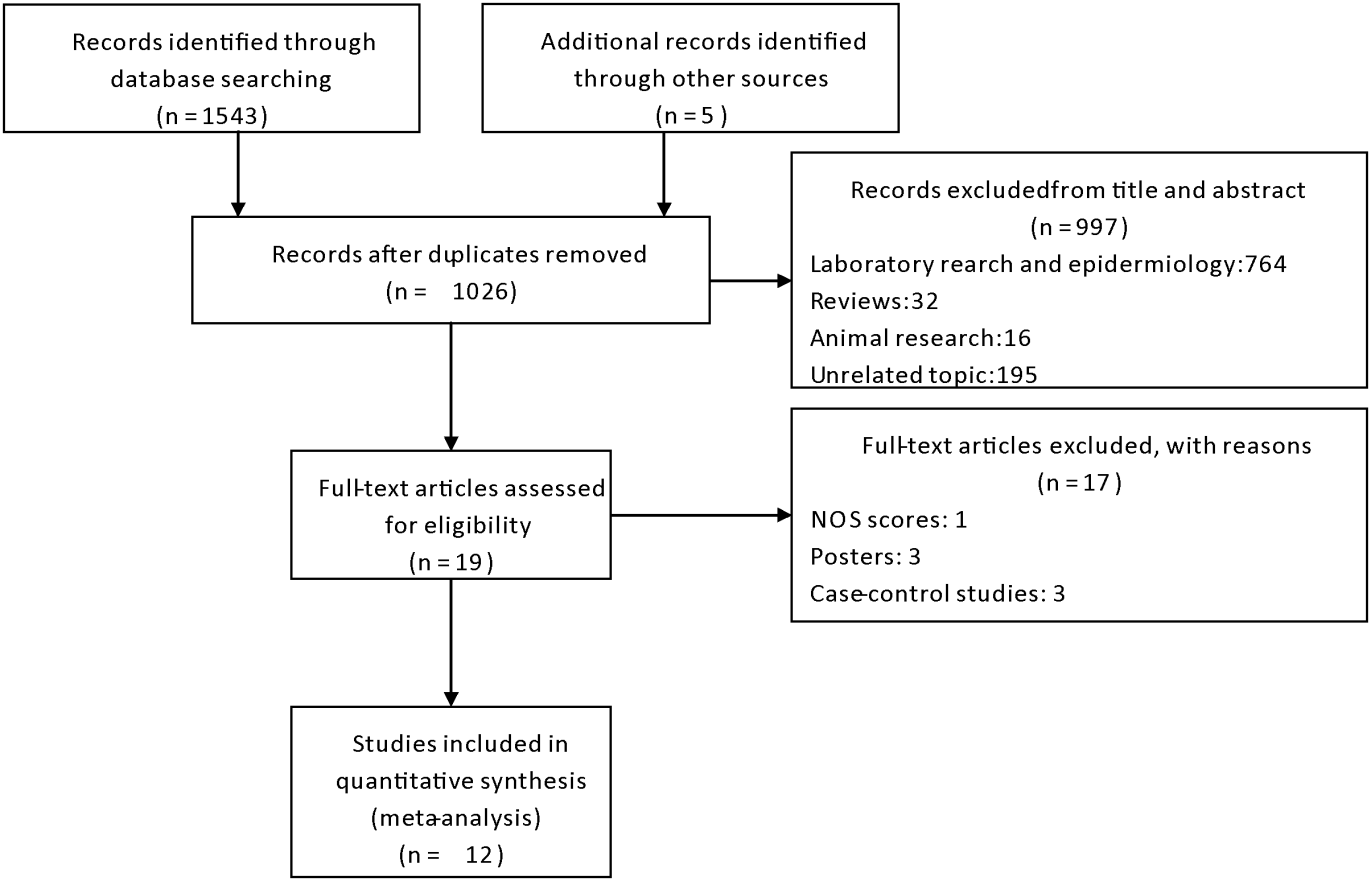
Flow diagram of included studies.

A total of 1548 articles were initially identified. The titles and abstracts were reviewed to exclude the irrelevant studies. Subsequently, 19 articles with full texts that met the inclusion criteria were assessed. One study was excluded for NOS score lower than 3. Three posters and three case-control studies were also excluded from the analysis. Hence, a final total of 12 studies were included in this meta-analysis. Figure 1 provides a flow diagram of the search.

### Study Characteristics

12 studies involving 677 participants satisfied the eligibility criteria for this meta-analysis. These studies included three RCTs, two prospective cohort studies and eight retrospective cohort studies. Eight studies compared polymyxins with other antibiotics and those comparator drugs included: ampicillin/sulbactam, cefoperazone/sulbactam, ceftazidime+amikacin, ceftazidime, cefepime, tobramycin, minocycline, doxycycline, tigecycline, doripenem, imipenem-cilastatin, meropenem, meropenem+piperacillin/tazobactam+vancomycin, doripenem+ampicillin/sulbactam. The remaining four studies compared polymyxins monotherapy with combination treatment and polymyxins were combined with rifampicin, sulbactam, cefoperazone/sulbactam, ampicillin/aulbactam, minocycline, cefoperazone/sulbactam+minocycline, cefoperazone/sulbactam+trimethoprim/sulfamethoxazole. Characteristics of the studies included in the analysis are presented in Table1.

**Table 1:** Characteristics of Studies Included in Systematic Review and Meta-analysis.

Author(Year)	Country	Type ofstudy	Experimentalgroup	Route ofPolymyxins	Sample size(Experimentalgroup/Controlgroup)	Type ofinfection	Organismsisolated	Age (Experimentalgroup vsControl group)	Sex (male/female)
Betrosian(2008) [20]	Greece	Prospectivecohort	Colistin	intravenous	15/13	VAP	MDRAB	67±9 vs 72±5(years)	14/14
Chan(2010) [23]	USA	Retrospectivecohort	Polymyxin Bor colistin	nebulized;intravenous;nebulized+intravenous	9/46	VAP	CRAB	40 (15–87)(years)	40/15
Garnacho(2003) [19]	Spain	Prospectivecohort	Colistin	intravenous	21/14	VAP	AB	56.9±13.1 vs 64.5±11(years)	26/9
Nakwan(2011) [24]	Thailand	Retrospectivecohort	Colistin	nebulized	8/7	VAP	EDRAB	38 (28–41) vs 29(28–34) (weeks)	10/5
Shields(2012) [25]	USA	Retrospectivecohort	Colistin	intravenous	32/5	VAP,VAT,Primarybacteremia	EDRAB	56 (21–80) (years)	26/15
Chia-Hao(2013) [26]	Taiwan	Retrospectivecohort	Colistin	nebulized	8/23	VAP	AB	29.60±3.93 vs29.17±2.92 (years)	13/18
Gounden(2009) [27]	SouthAfrica	Retrospectivecohort	Colistin	intravenous	32/32	BSI,RTI,SSII,meningitis,CRI,UTI	MDRAB	43.5±15.6 vs45.6±18.2 (years)	NS
Holloway(2006) [28]	USA	Retrospectivecohort	Polymyxin B	intravenous	33/4	VAP,BSI,UTI,SSI	MDRAB	41 (15–77) (years)	8/29
Aydemi(2013) [29]	Turkey	RCT	Colistin	intravenous	22/21	VAP	CRAB	61±20 (years)	30/13
Durante-Mangoni(2013) [30]	Italy	RCT	Colistin	intravenous	105/104	HAP, VAP,BSI,CIAI	EDRAB	61±15.7 vs62±15.1 (years)	137/72
Kalin(2013) [31]	Turkey	Retrospectivecohort	Colistin	intravenous	47/35	VAP	MDRAB	52 (19–96) vs63 (20–89) (years)	54/25
Jang(2009) [32]	Korea	Retrospectivecohort	Colistin	intravenous	22/19	VAP	MDRAB	62.5±17.5 vs57.0±16.5 (years)	25/19

Abbreviation: RCT = randomized controlled trial; VAP = ventilator associated pneumonia; VAT = ventilator associated tracheobronchitis; BSI = bloodstream infection; RTI =  respiratory tract infection; SSII =  skin or soft issue infection; CRI = catheter-related infection; UTI = urinary tract infection; SSI = surgical site infection; CIAI = complicated intra-abdominal infection; HAP = healthcare associated pneumonia; MDRAB = multi-drug resistant *Acinetobater baumannii*; EDRAB = extensively drug-resistant *Acinetobater baumannii*; CRAB = carbapenem-resistant *Acinetobacter baumannii*; AB = *Acinetobater bauma.*

### Methodological Quality

Two RCTs were evaluated by Jadad scale and both well reported randomization, concealment of allocation and withdrawal. All the NOS score of cohort studies achieved higher than 3.

### Statistical Results

#### Clinical response

**Figure 2:**
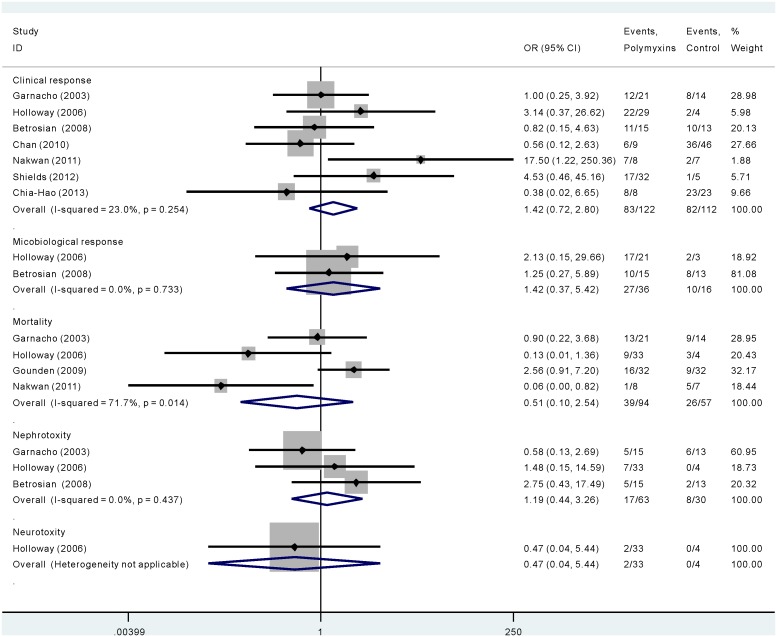
The efficacy and safety of polymyxins compared with other antibiotics in *Acinectobacter baumannii* infection.

As is shown in Figure 2, seven studies involving 234 participants compared polymyxins with other antibiotics in terms of clinical response. No statistical heterogeneity was observed among studies (χ^2^ = 7.8, *p* = 0.254, I^2^ = 23.0%). Although clinical response rates favored the polymyxins group (OR 1.421, 95% CI 0.722–2.797), the difference was not significant.

**Figure 3:**
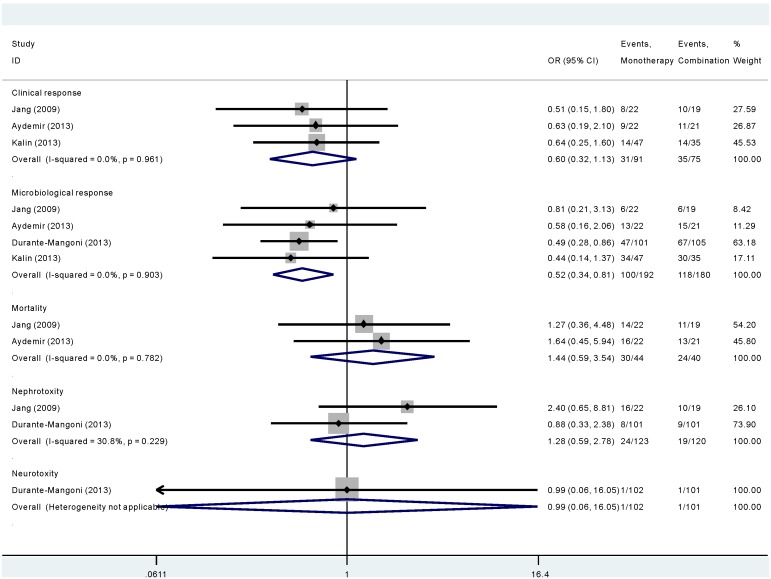
The length of stay in hospital of patients when polymyxins were compared with other antibiotics in *Acinectobacter baumannii* infection.

As is shown in Figure 3, three studies involving 166 participants compared polymyxins monotherapy with combination treatment in terms of clinical response. The overall clinical response did not differ significantly between monotherapy group and combination treatment group (OR 0.601, 95% CI 0.320–1.130). No statistical heterogeneity was observed among studies (χ^2^ = 0.08, *p* = 0.961, I^2^ = 0.0%).

#### Microbiological response

As is shown in Figure 2, of the two studies that reported the microbiological response of polymyxins compared with other antibiotics, no significant difference was found (OR 1.416, 95% CI 0.369–5.425). No statistical heterogeneity was observed among studies (χ^2^ = 0.12, *p* = 0.733, I^2^ = 0.0%).

As is shown in Figure 3, of the four studies comparing polymyxins monotherapy with combination treatment, there was a tendency for better microbiological response in polymyxins monotherapy group (OR 0.520, 95% CI 0.335–0.807). No statistical heterogeneity was observed among studies (χ^2^ = 0.57, p = 0.903, I^2^ = 0.0%).

#### Mortality

As is shown in Figure 2, for four studies that comparing polymyxins with other antibiotics, no significant difference was noted with respect to the hospital mortality (OR 0.506, 95% CI 0.101–2.536). There was statistical heterogeneity among studies (χ^2^ = 10.59, *p* = 0.014, I^2^ = 71.7%). Therefore, random-effects model of analysis was used.

As is shown in Figure 3, for two studies that comparing polymyxins monotherapy with combination treatment, no significant difference was noted with respect to the hospital mortality (OR 1.441, 95% CI 0.587–3.540). No statistical heterogeneity was observed among studies (χ^2^ = 0.08, *p* = 0.782, I^2^ = 0.0%).

#### Length of stay

**Figure 4:**
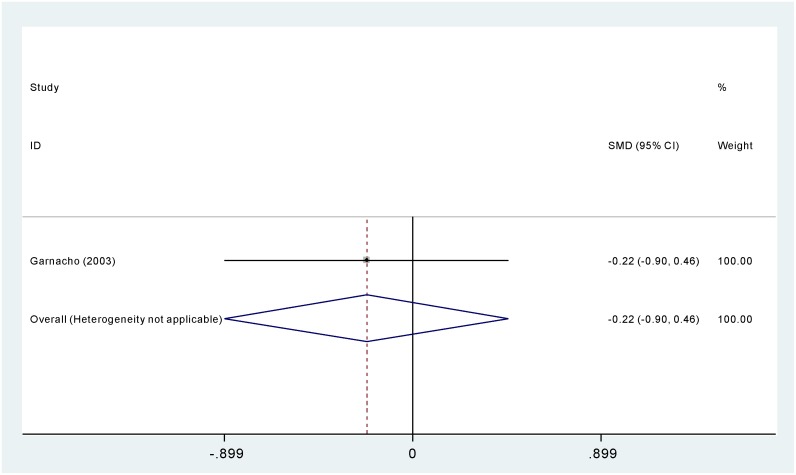
The efficacy and safety of polymyxins monotherapy compared with combination treatment in *Acinectobacter baumannii* infection.

As is shown in Figure 4, one study compared polymyxins with other antibiotics in terms of length of hospital stay. No significant difference was found between the two groups (SMD −0.221 days, 95% CI −0.899–0.458 days).

#### Adverse events

As is shown in Figure 2, when comparing polymyxins with other antibiotics administered intravenously in the nephrotoxicity in three studies, there was no significant difference between the two groups (OR 1.192, 95% CI 0.436–3.261). No statistical heterogeneity was observed among studies (χ^2^ = 1.66, *p* = 0.437, I^2^ = 0.0%). Both studies that administered polymyxins in nebulized route didn’t involve in nephrotoxicity. One study reported neurotoxicity. There was no significant difference between the two groups (OR 0.47, 95% CI 0.04–5.44).

As is shown in Figure 3, when comparing polymyxins monotherapy with combination treatment in nephrotoxicity in two studies, there was no significant difference between the two groups (OR 1.276, 95% CI 0.586–2.779). No statistical heterogeneity was observed among studies (χ^2^ = 1.44, *p* = 0.229, I^2^ = 30.8%). Only one study reported neurotoxicity, there was no significant difference between the two groups (OR 0.99, 95% CI 0. 0.06–16.05).

#### Publication bias

**Figure 5:**
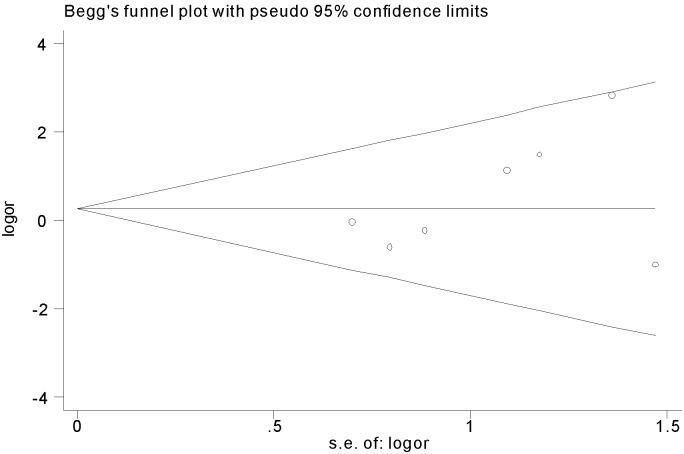
The funnel plot of clinical response rate when polymyxins were compared with other antibiotics in *Acinectobacter baumannii* infection.

There were no evidences of publication bias when Begg’s test (z = 0.90, p = 0.368) and Egger’s test were used (t = 1.38, p = 0.225). Therefore the funnel plot for publication bias demonstrated no marked evidence of asymmetry, which is shown in Figure 5.

## Discussion

*A. baumannii* has emerged as an increasingly common pathogen of healthcare associated infections in the intensive care unit [33], [34] and worldwide outbreaks have been reported recently [35]. Infection with this organism is associated with a high mortality rate and prolonged hospitalization [36], [37]. *A. baumannii* has the characteristics of rapid development of resistance to all the commonly used antibiotic classes, including the antipseudomonal penicillins, monobactams, carbapenems, aminoglycosides and quinolones [38]. In this situation, alternative options are scarce. A recent meta-analysis conducted by Chu et al. proved that sulbactam-based therapy exhibited almost no superiority to alternative antimicrobial therapies for the treatment of *A. baumannii* infection [39]. The ongoing studies for newer tigecycline also yielded controversy [40], [41]. Excitingly, the emerging therapeutic gap may be counterbalanced by the reuse of polymyxins to some extent [42]. In vitro, polymyxins usually rank first in antibacterial activity against MDR *A. baumannii* strains [43]. However, clinical studies are limited and did not always correlate with studies in vitro [42]. While awaiting the development of new antibiotics against MDR *A. baumannii*, it is of great necessity to assess the old antibiotics-polymyxins objectively.

To our knowledge, this is the first meta-analysis to compare the efficacy and safety of polymyxins with other antimicrobial agents for the treatment of *A. baumannii* infection. This meta-analysis executed an exhaustively search strategy without language restrictions, which may lead to a comprehensive analysis with less bias.

Owing to limited and controversial clinical studies were conducted concerning the efficacy of polymyxins in the treatment of *A. baumannii,* clinical doctors may hesitate in adopting this kind of antibiotics, which further intensify the unavailability of this promising drug in most regions. However, our meta-analysis may provide an encouraging result. In our study, all the efficacy outcomes including clinical response rate, microbiological response rate, hospital mortality, lengths of hospital stay are in favor of the polymyxins group without exception, although the difference was not statistically significant. We can at least conclude that polymyxins can be adopted as second-line therapy when the *A. baumannii* turned to be multi-resistant with little choice.

Our results also showed that treatment with polymyxins vs. controls did not affect nephrotoxicity, which may be the most concern of clinical doctor when making a choice about polymyxins. The reported incidence of colistin-associated nephrotoxicity reported ranged from 14% to 50% predating 1970 [44]–[47]. Recent data, however, has demonstrated less prominent nephrotoxicity than previously thought [44]. Despite commonly (48%) combined with aminoglycosides, colistin-related nephrotoxicity was developed in only 11% of patients in Pintado’s study [48]. Notably, in another two recent studies, no episode of renal insufficiency was observed in patients treated with colistin [49], [50]. The observed discrepancies between previous and current reports may be attributed to improvement in supportive treatment, close monitoring of renal function, lower doses, and purified formulation of polymyxins. It is reassuring that most of polymyxins-associated nephrotoxicity reported to date were reversible. In spite of this, we can’t be too optimistic. Recently, Chan et al. [51] reported a 57% incidence of colistin-related nephrotoxicity in the treatment of *A. baumannii* infection, a rate substantially higher than contemporary data. Neurotoxicity is also a common side effect of polymyxins with reported incidence ranging from 7% to as high as 29% in patients with cystic fibrosis [44]. We found no significant increase of neurotoxicity in the polymyxins group. As most included studies comprised of critically ill patients with high requirement of opiates and sedatives, it was difficult to assess neurological status attributed by polymyxins [23]. All in all, the safety of polymyxins therapy requires further investigation, and caution and close monitoring is of necessity.

Although exhibiting potent antimicrobial activity against *A. baumannii*, even polymyxins cannot escape resistance [51]. As a result, the use of combination regimens has been promoted other than a dosage increase regimen which may confer higher toxicity rates. Moreover, polymyxins monotherapy tends to be the potential cause of heteroresistance among patients exposed to polymyxins alone [52], [53]. Thus another rationale for employing combination regimen is to inhibit the heteroresistance. In fact, significant synergy has been observed in vitro when polymyxins are combined with rifampicin, ceftazidime, minocycline, or imipenem [54]. Whether these interactions are the same in vivo is undefined. The present research is also the first meta-analysis to assess the efficacy and safety of polymyxins monotherapy compared with combination therapy for the treatment of *A. baumannii* infection. Our study demonstrated that when compared with its combination regimen, polymyxins monotherapy regimen achieved similar clinical response rate, in-hospital mortality and length of stay in hospital. However, when it comes to microbiological response rate, we found that the combination regimen achieved better outcome. Currently, although there isn’t strong evidence that combination regimen is superior to monotherapy. Considering the better microbiological clearance, we need to do more clinical trials to screen out the potentially potent combination regimen.

Our study should be interpreted with caution, given certain limitations. Firstly, the sample size of this meta-analysis was small, which may reduce the power of statistical analysis. Secondly, some of our included controlled studies are not prospective RCTs, so we are unable to control some confounding factors. As an example, the outcomes of our meta-analysis were based on data pooled from studies of different durations, which was a compromise. Finally, owing to the limited number of studies, we didn’t put polymyxins B and colistin in different subgroups to analysis, which may otherwise provide more information.

In summary, the findings from our systematic review and meta-analysis suggest that polymyxins may be as safe and as efficacious as standard antibiotics for the treatment of *A. baumannii* infection. As there isn’t strong evidence that combination regimen is superior to monotherapy, we should be prudent when deciding whether to choose the combination regimen of polymyxins. However, given that the available evidence is limited, randomized, well-controlled clinical trials are needed to determine the role of polymyxins in treating *A. baumannii* infection.

## Supporting Information

Checklist S1 **PRISMA 2009 checklist.**
(DOC)
